# The Many Faces of Neuroendocrine Differentiation in Prostate Cancer Progression

**DOI:** 10.3389/fonc.2014.00060

**Published:** 2014-03-25

**Authors:** Stéphane Terry, Himisha Beltran

**Affiliations:** ^1^U955, Institut Mondor de Recherche Biomédicale, INSERM, Créteil, France; ^2^UMR 3244, Institut Curie, Paris, France; ^3^Division of Hematology and Medical Oncology, Weill Cornell Medical College, New York, NY, USA

**Keywords:** prostate cancer, neuroendocrine differentiation, small-cell carcinoma, cancer biology, NRSF/REST, androgen receptor, aurora kinase A, protocadherin

## Abstract

In normal prostate, neuroendocrine (NE) cells are rare and interspersed among the epithelium. These cells are believed to provide trophic signals to epithelial cell populations through the secretion of an abundance of neuropeptides that can diffuse to influence surrounding cells. In the setting of prostate cancer (PC), NE cells can also stimulate surrounding prostate adenocarcinoma cell growth, but in some cases adenocarcinoma cells themselves acquire NE characteristics. This epithelial plasticity is associated with decreased androgen receptor (AR) signaling and the accumulation of neuronal and stem cell characteristics. Transformation to an NE phenotype is one proposed mechanism of resistance to contemporary AR-targeted treatments, is associated with poor prognosis, and thought to represent up to 25% of lethal PCs. Importantly, the advent of high-throughput technologies has started to provide clues for understanding the complex molecular profiles of tumors exhibiting NE differentiation. Here, we discuss these recent advances, the multifaceted manner by which an NE-like state may arise during the different stages of disease progression, and the potential benefit of this knowledge for the management of patients with advanced PC.

## Introduction

Prostate cancer (PC) is the most frequently diagnosed malignancy in men and is accompanied by a frequent source of morbidity and mortality (1). The vast majority of prostate tumors appear in the form of adenocarcinoma, a type of tumor assumed to derive from transformation of the glandular cells of the prostate. Like the normal prostatic glands that develop, regenerate, and function under the influence of androgens, prostatic adenocarcinoma (thereafter adeno-PC) also relies on androgens working through the androgen receptor (AR) for its development and progression. This is the basis for the use of therapeutic intervention to block androgen synthesis (e.g., chemical and surgical castration), or inhibit AR function (e.g., AR antagonists), as standard therapies for patients with advanced and recurrent diseases (2, 3). Although these treatments provide for most patients clinical responses and symptomatic relief, explained partly by an effective blockade of proliferation and induction of cell death in a proportion of PC cells, they rarely eradicate all PC cell populations, and ultimately most patients develop resistance to these treatments as well as an almost uniformly fatal development. For many scientists and clinicians, the manifestation of “neuroendocrine differentiation” or a “neuroendocrine phenotype” is associated with progression of PC to castrate-resistant disease generally representing late-stage and lethal events in prostate tumorigenesis. However, these generic terms assume many different aspects and components during natural or treatment history of PC that are not completely understood due to lack of knowledge of the underlying biology. The intent of this review is to present our current knowledge about the multifaceted forms that can take neuroendocrine (NE) differentiation in PC and describe recent advances in our understanding of the molecular perturbations that might control or drive the NE phenotype in this disease.

## Neuroendocrine Cells of the Human Prostate

Neuroendocrine cells are found in many tissue types including normal prostate. The prostate is a male accessory sex gland that functions to produce a major fraction of seminal fluid. In normal prostatic parenchyma, the NE cells are generally rare and widely interspersed amongst the epithelial basal and luminal cells grouped within glandular structures called prostatic acini and ducts (4). As part of a diffuse NE system, prostatic NE cells secrete many types of neuropeptides (including bombesin, calcitonin, parathyroid-like hormone, serotonin, and adrenomedullin) and growth factors (including VEGF) that are believed to maintain homeostasis of the surrounding epithelial cell populations (5). In the cancer setting, they can persist, and their secretory products may influence surrounding PC cells by acting on their rates of cell death and/or proliferation (5–7). Prostatic NE cells present with dendritic processes comparable to that found in neuronal cells and express a variety of NE markers (including chromogranins, synaptophysin, and CD56). These cells may derive from the differentiation of progenitors located in the basal cell layer. As terminally differentiated cells, they are devoid of proliferative activity and usually express anti-apoptotic factors such as survivin (8). Further research is needed to decrypt the role of normal NE cells in the physiopathology of PC. Intriguingly, their number may vary depending upon the ethnic backgrounds. They are found at a relatively low rate in African-American men, a population more likely to develop PC, which supports a protective role for this cell type in prostate carcinogenesis (9).

## *De Novo* Neuroendocrine Prostate Tumors

An extremely small proportion of PC patients present with overt “*de novo*” NE tumors in that these tumors are composed of NE tumor cells and diagnosed outside the context of a previously known adeno-PC. Immunohistochemical examination usually shows negativity for AR as well as for prostate specific antigen (PSA), and positivity for NE markers with varied levels. NE tumors include small-cell carcinoma of the prostate (SCPC) (<0.1% of all diagnosed PCs) (10) as well as exceptionally rare tumors such as large cell NE carcinoma (a few cases worldwide) (11), and low-grade carcinoid, comparable to carcinoid in other locations (12). These tumors can be pure or admixed with prostatic adenocarcinomas. An atypical situation has been described in one case of a so-called “hybrid” tumor presenting with both NE and epithelial phenotypes (13).

Small-cell carcinoma of the prostate has been described by numerous investigators and is defined as a poorly differentiated NE cancer microscopically identical to its most common counterpart in the lung, small-cell lung carcinoma (14, 15). Unlike the scattered NE cells in normal or benign prostate, the NE tumor cells in SCPC are highly proliferative, metastatic, and resistant to most conventional therapies. As a result, SCPC is invariably fatal with most patients dying within 2 years of diagnosis despite very aggressive chemotherapeutic regimens (16–18). To date, the origin of these tumors remains uncertain. They can share similarities with basal/progenitor cells (P63^+^, c-Kit^+^) (19). This has led to the speculation that they derive from transformation of multipotential prostatic progenitors. Other studies performed in genetically engineered mouse models have suggested the transformation of normal prostatic NE cells as origin of these tumors (20). Finally, as will be repeatedly reiterated in this review, ample evidence pleads in favor of the transformation of adenocarcinoma.

## Neuroendocrine Differentiation in Untreated Prostate Adenocarcinoma

Hormonally untreated adeno-PCs, sometimes referred to as “hormone naïve” adeno-PCs, also contain isolated cells staining positive for NE markers. In most cases, these cells are present in smaller numbers compared with that in normal or benign prostate. However, in 5–10% of adeno-PCs, these cells are found in greater numbers in the form of solitary cells, or aggregates of densely packed cell clusters (<50 cells). This pattern is designated as focal NE differentiation and the adenocarcinoma contingent remains predominant in such setting. Moreover, these cells are usually referred to as “NE-like” cells as they do not necessarily resemble normal NE cells but rather show the same appearance as adenocarcinoma cells (21). Importantly, genetics characterization of laser captured microdissected cells from pathological specimens has demonstrated that these NE-like cells are malignant cells linked genetically to the neighboring adenocarcinoma cells (22). This knowledge, also supported by number of experimental data generated *in vitro* and in rodents, led to the notion that prostate adenocarcinoma cells have the capacity to transdifferentiate to acquire a more NE phenotype through a process termed NE transdifferentiation. It has been suggested to play a role in sustaining prostate tumor growth and progression (23, 24). Yet, controversy arises especially when interrogating for prognostic utility of assessing for NE markers in tissues or in blood (25, 26). Other clinical studies have found that pretreatment NE differentiation may have prognostic utility in subgroups of patients being treated for advanced disease. In a population of patients who received radiotherapy as a primary treatment, the presence of NE differentiation involving >1% of tumor cells on initial PC biopsies has been associated with an increased risk of distant metastasis and reduced cancer-specific survival time (27, 28). Evaluation of independent patient cohorts may be needed to determine the veracity of these new findings.

On the other hand, it is well-accepted that cultured PC cells can be directly induced to undergo an NE transdifferentiation process *in vitro* by exposure to a wide spectrum of stimuli. This phenotypic switch has been seen especially in cultures of the PC cell line LNCaP, and occasionally in cultured PC3 and DU145 cell lines, when cells are grown in medium supplemented with dibutyryl-cyclic AMP (db-cAMP) (29), forskolin, cytokines (e.g., IL6), growth factors (e.g., EGF, HB-EGF), or in hormone-depleted medium (30). This topic has been extensively covered by two reviews to which the reader is referred (31, 32). It is important to consider that some of these factors may be components of the tumor microenvironment in some patients prior to any therapeutic intervention. However, this focal NE differentiation appeared to be relatively uncommon in prostate tumors of patients who did not receive any systemic therapy, and hence, NE transdifferentiation is becoming increasingly recognized as an adaptation mechanism allowing PC cell populations to adapt a wide spectrum of therapeutic agents (33–36).

## Neuroendocrine Differentiation in Hormonally Treated and Castrate-Resistant Prostate Cancers

There seems to be a consensus, among PC researchers, to say that transdifferentiation from an epithelial-like phenotype to an NE-like phenotype represents a noteworthy biological process that can be considered a consequence of the selective pressure induced by all treatments that lead to a fall in androgen levels, or to blocking the action of these steroid hormones. Ralph Buttyan and co-workers were the first to observe that in LNCaP cells, which is by far the most commonly used model in PC research (37), the cells undergo an NE transdifferentiation, when chronically exposed to medium lacking androgens and that restoring androgens back to the medium suppressed this NE transdifferentiation state (30). Many laboratories have confirmed this finding, as well as showing that in most instances the transdifferentiation implicates a reduced activity and/or expression of AR (38). Additionally, there have been reports in clinical literature that focal NE differentiation is increased within castrate-resistant PC (CRPC) tumors as well as levels of NE-derived peptides such as neuron-specific enolase (NSE) and chromogranin-A in the serum of CRPC patients (26, 39, 40). In patient specimens, this is usually reflected by an increase in the size and number of overt NE-like clusters (36, 39, 41). These reports also showed that the increased presence of the NE component can manifest a few months following implementation of androgen deprivation therapy, which may be months or even years before the diagnosis of castrate resistance. This data coupled with clinical observation that adenocarcinoma cells present in the prostate are generally slowly proliferating (42), again support the idea that NE-like tumor cells have transdifferentiated from adenocarcinoma in response to treatment. One important question has remained unsolved: does these coexisting tumor variants interact with each other “*in vivo*” as a means for progression? First attempts to address this question indicate this might be the case (43, 44). Additional studies are needed to validate this hypothesis.

Moreover, one must consider the possible metaplastic nature of NE-like tumor cells. In this respect, some evidence already denotes that NE differentiation may take various forms during tumor progression with NE-like tumor cells exhibiting diverse NE traits, sometimes with loss or gain expression of certain NE markers (36, 38, 41, 45, 46). NE transdifferentiation may be partial, incomplete, or even reversible. A similar situation applies with other types of transdifferentiation processes (EMT or MET for example) (47). Thus, we posit that under prolonged hormonal manipulations, multiple NE-like tumor cell populations, or NE subtypes (from well to poorly differentiated), be selected within cancers of varied proliferative activity, expressing various levels of NE markers and/or more epithelial markers. In tumors having progressed to a therapeutic resistance, NE state may transiently disappear to reappear later on with subsequent treatments administered including anti-AR or chemotherapy regimens.

Importantly, a substantial proportion of heavily treated CRPC shows many of the salient features of *de novo* small-cell carcinomas, admixed or not with adenocarcinoma. It is currently difficult to assess the prevalence of such SCPC disease because metastatic CRPC patients are not routinely biopsied in end-stage disease. Nevertheless, it is estimated that at least 25% of patients with advanced PC may eventually develop this type of highly aggressive NEPC (48). In some cases, SCPC not only coexists with an adenocarcinoma component, but also shares genetic alterations such as *TMPRSS2–ERG* gene fusion (49–51) that are thought to arise specifically from prostatic adenocarcinoma cells responding to androgens (52, 53). This again is evocative of a transdifferentiation model to explain the emergence of this NEPC type. Alternatively, this could indicate that small-cell carcinoma and adenocarcinoma components may arise from a tumor clone with stem-like properties that gave rise to at least two distinct components (i.e., epithelial and NE), which were able to persist and evolve in parallel during tumor progression. In cases of clinically suspected SCPC progression, patients are often treated with platinum-based chemotherapy with regimens similar to small-cell lung cancer (54, 55). However, despite initial responses, most patients progress rapidly and there is no standard second line therapy.

We and others are concerned that with the introduction of novel potent AR-targeted drugs into the clinic for CRPC, the incidence of SCPC transformation may increase (48). Future investigations should address these questions, but not restrict the analysis to anti-AR targets. We already know that cultures of androgen-independent LNCaP derivatives, apparently refractory to NE transdifferentiation caused by an androgen impoverished environment, can regain numerous NE attributes under chronic exposure to docetaxel (36), the standard-of-care first-line chemotherapy for CRPC patients. Whether such finding can be transferred to patients or any other preclinical models has yet to be documented. Additionally, several pieces of evidence suggest that ionizing radiation induces NE transdifferentiation of LNCaP cells with the acquisition of cross-resistance to radiotherapy, chemotherapy, and hormonal therapy (33). It will be important to further investigate this intriguing possibility in clinical studies, especially because many PCa patients receive radiotherapy either as a primary therapy, salvage therapy, or in combination with surgery or hormonal therapy (56, 57). Of note, Komiya and colleagues have recently reported a case of SCPC, which developed after high-dose-rate brachytherapy (58).

These instances illustrate the complexity of the molecular events that govern NE differentiation and support the need to identify novel molecular components and markers that may assist in deciphering NE transdifferentiation process and better classify the different grades of NE differentiation as well as their significance.

## Toward Molecular Understanding of What Drives Neuroendocrine Phenotype in Prostate Cancer

The underlying biology of NEPC has remained a conundrum for scientists and clinicians alike. A better understanding of the molecular events underlying NEPC transformation is urgently needed to provide therapeutic solution in the management of PC patients with lethal diseases. The recent discovery of some of the molecular components and genetic alterations driving the NE phenotype in PC takes up an important step closer to this goal. Using next-generation sequencing as a tool to define the transcriptome of both NE- and adeno-PCs, we discovered that the vast majority of NEPC over-express the cell cycle kinase *AURKA* (Aurora kinase A) and *MYCN* compared to adenocarcinoma (50). These two proto-oncogenes cooperate to drive a malignant phenotype similar to other N-myc driven tumors (59). Gene amplification could explain the high-level expression of these genes in most instances. Moreover, these two proto-oncogenes seem to work in synergy to drive aggressiveness and NE phenotype in PC. Perhaps more importantly, in this study we provided preclinical evidence that inhibition of Aurora kinase using PHA-739358 treatment effectively impeded the growth of NE tumor cells *in vitro* and *in vivo*. This observation stimulated the implementation of a clinical trial that will evaluate the effectiveness of a second-generation inhibitor of Aurora kinase A in patients with metastatic PCa with confirmed NE phenotype, or strong suspicion of NE disease (metastatic development in the absence of PSA progression) (ClinicalTrials.gov; identifier: NCT01799278).

Notably, in our original study it was also possible to detect AURKA over-expression and amplification in a few primary adeno-PC cases, at least in a small fraction of neoplastic cells (50). An important follow-up question has been to determine if adeno-PC cases harboring *AURKA* and/or *MYCN* alterations are more likely to transform into aggressive NEPC. To address this point, we have inspected for *AURKA* and *MYCN* alterations in tissue specimens from selected patients having developed NEPC along the course of their disease, comparing it to an unselected series with no apparent history of NE-related disease (60). This retrospective assessment showed that *AURKA* and *MYCN* amplifications in primary adeno-PC effectively predict for a late-stage development of NEPC in CRPC patients also suggesting these molecular alterations may predispose for development of the small-cell disease from adeno-PC.

Recent surveys suggest that RB loss (61–63) and MYC over-expression underlie the development of NEPC lesions (16, 64). Perturbations of the tumor microenvironment, including hypoxic conditions also appeared to play a role in the emergence of an NE phenotype (65, 66). In 2010, Ronai and his colleagues reported on the potential role of a hypoxia-mediated “FoxA2/HIF-1a” complex in driving NE phenotype in mouse prostate tumors, which is concordant with previous studies pointing out the preferential occurrence of FOXA2 expression in human NEPC (67). If confirmed, these results could provide a biologic rationale for further assessment of targeting these components to prevent NEPC development.

Another significant breakthrough came from the study of Lapuk et al. with the discovery of REST downregulation in association with the prevalence of NE phenotype in PCa (68). It is relevant to note here that a recent survey suggests that hypoxia induces miR-106b, miR-93, and miR-25, which may in turn down-regulated REST in PC cells (69). REST namely RE1-silencing transcription factor [also known as neuron-restrictive silencer factor (NRSF)] was identified in 1995 as a master repressor of the neuron-specific acting genes during neurogenesis but it very rapidly emerged that REST plays a broader role than originally anticipated (70, 71). In LNCaP cells, REST depletion resulted in upregulation of several NE markers (68). In their 2013 study, Svensson et al. confirmed this information and revealed novel molecular paradigms linking the androgen/AR axis, REST, and NE differentiation (72). Also intriguing was the discovery of an invert correlation between REST and the protocadherin (PCDH) genes *PCDH11Y* and *PCDH11X*.

Protocadherins constitute the largest subfamily of cadherins in the genome. PCDH genes are overwhelmingly expressed in neuronal cells of the central and peripheral nervous systems where they perform important functions for formation, maintenance, and integrity of neural circuitry (73). Recently, they have received increased attention for their roles in cancer (74). *PCDH11Y* gene products are highly upregulated in hormonally treated and CRPC tumors and so are also commonly referred to as *PCDH–PC* (36, 75). In collaboration with Dr. Buttyan and his colleagues, we have shown that over-expression of PCDH–PC in human prostate adenocarcinoma cells promotes NE transdifferentiation and this is further supported by our survey of human specimens (36, 76). The human and male specific nature of this PCDH is particularly intriguing in the context of PC, a malignant disease that is mainly restricted to humans. *PCDH11Y/PCDH–PC* gene lies within human Y chromosome at Yp11.2 (77, 78). This genomic region was acquired during evolution from primates to humans from duplication–transposition of an X chromosome region [containing *PCDH11X* (Xq21.3)] onto the Y chromosome (79, 80). The latter likely evolved and acquired a few sequence changes that significantly altered translation products diverging functionally from those of PCDH11X products (77, 78, 80). To our surprise, Svensson et al. did not report on deregulation of other PCDH members following perturbation of REST expression, while previous surveys in vertebrates point to a universal role of REST in regulation of PCDH genes (81, 82). This argues for exclusive regulatory events in place in PC cells. Interestingly, the transcriptomic data obtained also indicate that REST not only acts to repress neuronal genes but also genes involved in cell cycle progression, including *AURKA*. Follow-up studies should help get better insight into this regulation. Another important aim will be to explore further if perturbations of REST in PC cells can regulate other PCDH members with potential effect in directing the NE phenotype.

## Integrating Neuroendocrine Differentiation in the Context of Heterogeneity and Plasticity of Prostate Cancer

Despite significant advances in the identification of stimuli and molecular events presumably governing NE differentiation, it is a utopia to think that these events take place in all PC cells. This is illustrated by the observation that under some circumstances, over-expression of *PCDHY*/*PCDH–PC* seems capable of supporting tumor cell growth and resistance to cell death without any marked increase or association with NE differentiation (36, 75, 78). *In vitro*, PCDH–PC over-expression induces NE transdifferentiation of the LNCaP cells through a Wnt signaling-dependent mechanism, but in cell lines with a different genetic background, PCDH–PC appears to stimulate this pathway without apparent effects on the NE phenotype (published and non-published data). Whereas subsequent studies have substantiated the link between Wnt activation and NE differentiation in various PC models (83, 84), other studies have found Wnt activation as being associated with self-renewal (85), oncogenesis, and castration resistance (86, 87). These disparate effects can be explained by clonal-dependent response of cells for a given signal. In fact, there is no doubt that NE differentiation and NE transdifferentiation, like for many other biological processes, are largely influenced by the origin of the cells as well as by clonal oncogenic events probably emerging during tumor development. It is well-established that aberrant Wnt signaling is involved in various malignancies and that Wnt signaling is also important for differentiation and normal development, including neural development (88, 89).

Like Wnt signaling, REST appears to possess different activities in various tumor or cellular contexts (90). REST expression may have pro-oncogenic activity in nervous tumors and is found consistently upregulated in neuroblastomas and medulloblastomas (91, 92), while in small-cell lung carcinomas, loss of REST activity is observed and related to NE phenotype (93). In neural stem/progenitor cells, REST can cooperate with other molecular alterations including MYC over-expression to induce tumor formation in the cerebellum by blocking neuronal differentiation and maintaining the “stemness” of these cells (94). Little is known about how developmental signaling pathways influence REST function. This is an area in which more research is needed. In the case of PC, investigators will have to deal with the multiple nodes through which these pathways (e.g., Wnt, hedgehog, and Notch) can influence AR signaling at the different stages of PC progression (66, 95–98). Androgen/AR axis exerts a protective effect toward REST in adeno-PC (72). Since aberrant Wnt signaling can modulate AR signaling at multiple levels and trigger NE transdifferentiation, it is tempting to speculate that Wnt signaling also plays some role (direct or indirect) in REST regulation. Of further interest, there are reasons to believe that canonical Wnt pathway directly regulates the expression of REST (99), and that REST cross-talks with hedgehog signaling (100). In this line, Wnt and Hedgehog pathways have been implicated in pulmonary and extra-pulmonary small-cell carcinomas (101–103). Furthermore, FOXA2 gene, a gene critically involved in NEPC tumors (65, 67), is a direct target of hedgehog signaling (104) and is induced by active Wnt signaling (105).

**Figure 1:**
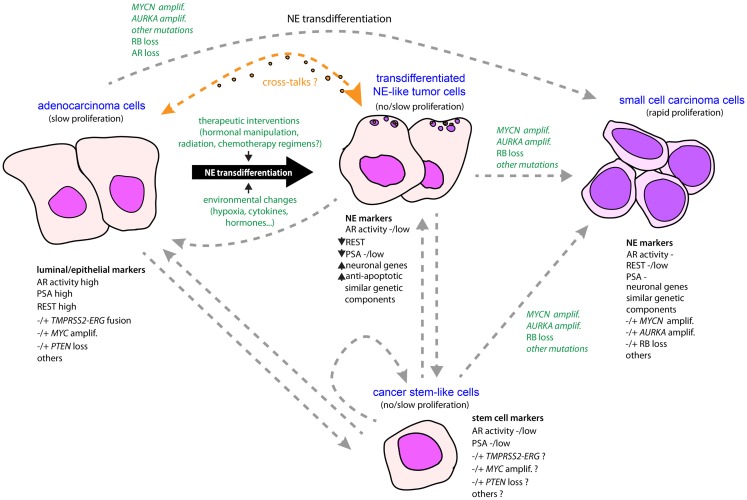
**Schematic model for the emergence of NE phenotype in tumor progression in prostate cancer**. An NE-like or NE phenotype may be driven by a conjunction of molecular events and environmental changes, combined with natural selection. Owing to treatment intervention and/or certain environmental changes associated with tumor development, some adenocarcinoma cells having accumulated genetic alterations are able to transdifferentiate from epithelial-like to neuroendocrine-like tumor cells that have superior anti-apoptotic and therapeutic-resistant properties. In addition, neuropeptides and growth factors released by the NE-transdifferentiated cells may act either as autocrine or paracrine signals to promote survival and tumor progression of both NE and adenocarcinoma components. Accumulation of new genetic alterations such as RB loss, *AURKA*, and *MYCN* amplifications under evolutionary pressures, or through natural selection of preexisting NE-like tumor subpopulations, may promote rapid cell expansion and subsequent emergence of a predominant NE component in the form of small-cell carcinoma characterized by rapid proliferative activity. Alternatively, small-cell carcinoma could arise by direct transformation of adenocarcinoma. Adenocarcinoma and small-cell carcinoma components may also originate from a common tumor clone with stem-like properties, or a cancer stem cell, converted into more differentiated tumor cells that accumulated molecular alterations driving epithelial or NE phenotypes. To date, very limited amounts of data are supportive of normal prostatic NE cell as a cell of origin for NE tumors (not shown here).

One pending question concerns the molecular similarities and differences between focal NE differentiation found in adeno-PC and extensive NE differentiation that dominates in SCPC, coexisting or not with adeno-PC. To what extent these are helpful to support or refute the process of NE transdifferentiation as origin of NEPC? As we now understand it, REST is essential in controlling NE phenotype of both SCPC and focal NE in adeno-PC. Potentially modulated by a long list of factors, it can be considered as a universal regulator of NE phenotype. However, the proliferative activity of NE tumor cells of SCPC generally contrasts with that observed in adeno-PC even when assessment is performed at advanced stage (106). Although the proliferation rate of adeno-PC certainly increases in the metastatic setting, adeno-PC is still considered as a slow progressing tumor type (42), and it seems likely that tumor cells confined into NE-like clusters are likewise slow-growing or quiescent (Figure 1). Nonetheless, in that situation, the peptide hormones produced by NE-like tumor cells may be feeding adjacent and even distant adenocarcinoma cells. Whether these adenocarcinoma cells are truly addicted to the NE component for their relatively “slow” but “persistent” growth is a matter of future investigations.

As noted above, genetic alterations such as *MYCN* and *AURKA* amplifications or RB depletion are considerably enriched in SCPC (50, 63). These alterations may be the manifestation of commitment through an uncontrolled proliferative state coinciding with the rapid tumor progression of this form of NEPC (Figure 1). Additional relevant alterations include *MYC* amplification, *TP53* mutation, or PTEN reduction, which are found in both SCPC and adeno-PC principally in late-stage disease (63, 107). Importantly, each of these events has the potential to sustain PC cell growth in androgen-deprived or AR-inactivated conditions (108–111). This is consistent with the seeming AR-independent nature of most if not all SCPC. Thus, although the direct role of these genetic perturbations in driving an NE phenotype is far from being established, it remains that they may participate in controlling proliferative activity and survival of SCPC. Likewise, if we assume that some of these molecular events occur in at least a fraction of quiescent NE-like tumor cells confined to adeno-PC, it is then easy to foresee how the proliferative activity will increase in these cells, and hence, facilitate expansion of the NE component in adeno-PC, that perhaps will become the predominant pattern in an AR-independent context (Figure 1). Further studies will be necessary to elucidate the precise molecular networks engaged downstream of these alterations in the different NE populations. The current development of single-cell purification techniques coupled with high-throughput analyses should provide valuable information toward this goal.

## Conclusion

Neuroendocrine cell-like tumor cells as well as pure NE tumor cells are present at all stages of prostate tumor development. Rarely predominant *de novo*, NE components are especially enriched in the late stages of the disease coincident with metastatic spread and treatment resistance. This NE enrichment may be explained by transdifferentiation of preexisting adenocarcinoma cells or the derivation of clones with stem-like properties differentiating into various phenotypes (Figure 1). In one way or the other, these two processes are preferentially mobilized under persistent treatments in conjunction with appearance of genetic perturbations initiating or maintaining an NE phenotype. The pattern of these molecular events may also explain why most NE-like tumor cells in adeno-PC remain quiescent when the vast majority of NE tumor cells in SCPC are rapidly cycling. On the other hand, the early occurrence of these events in adeno-PC could presage the emergence of SCPC from adeno-PC. In a sense, PC represents a unique situation among other malignancies in which the NE system is progressively mimicked by cancer cells and eventually corrupted so as to adapt and progress through treatments. While therapeutic strategies have focused on attempting to block the androgen/AR axis, targeting the NE component has the potential to provide new therapeutic solution in the management of PC patients with painful and lethal disease. Research and clinical studies are now underway to improve treatment modalities and test new agents that hold great promise in treating patients with this condition.

## Conflict of Interest Statement

The authors declare that the research was conducted in the absence of any commercial or financial relationships that could be construed as a potential conflict of interest.
